# Prevalence and Clinical Presentation of Health Care Workers With Symptoms of Coronavirus Disease 2019 in 2 Dutch Hospitals During an Early Phase of the Pandemic

**DOI:** 10.1001/jamanetworkopen.2020.9673

**Published:** 2020-05-21

**Authors:** Marjolein F. Q. Kluytmans-van den Bergh, Anton G. M. Buiting, Suzan D. Pas, Robbert G. Bentvelsen, Wouter van den Bijllaardt, Anne J. G. van Oudheusden, Miranda M. L. van Rijen, Jaco J. Verweij, Marion P. G. Koopmans, Jan A. J. W. Kluytmans

**Affiliations:** 1 Department of Infection Control, Amphia Hospital, Breda, the Netherlands; 2 Amphia Academy Infectious Disease Foundation, Amphia Hospital, Breda, the Netherlands; 3 Julius Center for Health Sciences and Primary Care, University Medical Center Utrecht, Utrecht University, Utrecht, the Netherlands; 4 Laboratory for Medical Microbiology and Immunology, Elisabeth-TweeSteden Hospital, Tilburg, the Netherlands; 5 Department of Infection Control, Elisabeth-TweeSteden Hospital, Tilburg, the Netherlands; 6 Microvida Laboratory for Medical Microbiology, Bravis Hospital, Roosendaal, the Netherlands; 7 Microvida Laboratory for Medical Microbiology, Amphia Hospital, Breda, the Netherlands; 8 Department of Medical Microbiology, Leiden University Medical Center, Leiden, the Netherlands; 9 Department of Virology, Erasmus Medical Center, Rotterdam, the Netherlands

## Abstract

**Importance:**

On February 27, 2020, the first patient with coronavirus disease 2019 (COVID-19) was reported in the Netherlands. During the following weeks, at 2 Dutch teaching hospitals, 9 health care workers (HCWs) received a diagnosis of COVID-19, 8 of whom had no history of travel to China or northern Italy, raising the question of whether undetected community circulation was occurring.

**Objective:**

To determine the prevalence and clinical presentation of COVID-19 among HCWs with self-reported fever or respiratory symptoms.

**Design, Setting, and Participants:**

This cross-sectional study was performed in 2 teaching hospitals in the southern part of the Netherlands in March 2020, during the early phase of the COVID-19 pandemic. Health care workers employed in the participating hospitals who experienced fever or respiratory symptoms were asked to voluntarily participate in a screening for infection with the severe acute respiratory syndrome coronavirus 2. Data analysis was performed in March 2020.

**Main Outcomes and Measures:**

The prevalence of severe acute respiratory syndrome coronavirus 2 infection was determined by semiquantitative real-time reverse transcriptase–polymerase chain reaction on oropharyngeal samples. Structured interviews were conducted to document symptoms for all HCWs with confirmed COVID-19.

**Results:**

Of 9705 HCWs employed (1722 male [18%]), 1353 (14%) reported fever or respiratory symptoms and were tested. Of those, 86 HCWs (6%) were infected with severe acute respiratory syndrome coronavirus 2 (median age, 49 years [range, 22-66 years]; 15 [17%] male), representing 1% of all HCWs employed. Most HCWs experienced mild disease, and only 46 (53%) reported fever. Eighty HCWs (93%) met a case definition of fever and/or coughing and/or shortness of breath. Only 3 (3%) of the HCWs identified through the screening had a history of travel to China or northern Italy, and 3 (3%) reported having been exposed to an inpatient with a known diagnosis of COVID-19 before the onset of symptoms.

**Conclusions and Relevance:**

Within 2 weeks after the first Dutch case was detected, a substantial proportion of HCWs with self-reported fever or respiratory symptoms were infected with severe acute respiratory syndrome coronavirus 2, likely as a result of acquisition of the virus in the community during the early phase of local spread. The high prevalence of mild clinical presentations, frequently not including fever, suggests that the currently recommended case definition for suspected COVID-19 should be used less stringently.

## Key Points

**Question:**

What was the prevalence and clinical presentation of coronavirus disease 2019 among health care workers with self-reported fever or respiratory symptoms in 2 Dutch hospitals within 2 weeks after the first patient with coronavirus disease 2019 was detected in the Netherlands?

**Findings:**

In this cross-sectional study that included 1353 health care workers with self-reported fever or respiratory symptoms, 6% were infected with severe acute respiratory syndrome coronavirus 2. Most health care workers with coronavirus disease 2019 experienced mild disease, and only 53% reported fever.

**Meaning:**

The high prevalence of mild clinical presentations, frequently not including fever, suggests that the currently recommended case definition for suspected coronavirus disease 2019 should be used less stringently.

## Teaser

This cross-sectional study examines the prevalence and clinical presentation of coronavirus disease 2019 among health care workers in the Netherlands with self-reported fever or respiratory symptoms.

## Introduction

Since December 2019, the world has been in the grip of the severe acute respiratory syndrome coronavirus 2 (SARS-CoV-2) and the disease it causes, coronavirus disease 2019 (COVID-19).^1^ On February 27, 2020, the first patient with COVID-19 was detected in the Netherlands, after a trip to northern Italy between February 18, 2020, and February 21, 2020.^2^ From then until March 6, 2020, another 127 COVID-19 cases were identified in the Netherlands, including 9 health care workers (HCWs) in 2 Dutch teaching hospitals in the southern part of the Netherlands who received a diagnosis of COVID-19 between March 2, 2020, and March 6, 2020. Eight of these 9 HCWs had no history of travel to China or northern Italy, raising the question of whether undetected community circulation was occurring. Because these findings coincided with the seasonal influenza peak^3^ and because SARS-CoV-2 infection in HCWs could lead to both sick leave and introduction of the virus into the hospital, this finding prompted a demand for testing HCWs. After initial observations of SARS-CoV-2 detection in persons with mild symptoms who did not meet the definition for case finding,^1^ screening for SARS-CoV-2 was implemented to determine the prevalence and the clinical presentation of COVID-19 among HCWs with self-reported fever or respiratory symptoms in these 2 hospitals.

## Methods

### Study Design, Setting, and Population

The study was reviewed by the Ethics Committee Brabant, the Netherlands. The study was judged to be beyond the scope of the Medical Research Involving Human Subjects Act, and a waiver of written informed consent was granted. Oral informed consent was obtained from all HCWs for SARS-CoV-2 testing and from SARS-CoV-2–infected HCWs for data collection. Data were deidentified before analysis. This study follows the Strengthening the Reporting of Observational Studies in Epidemiology (STROBE) reporting guideline.

**Figure 1.**
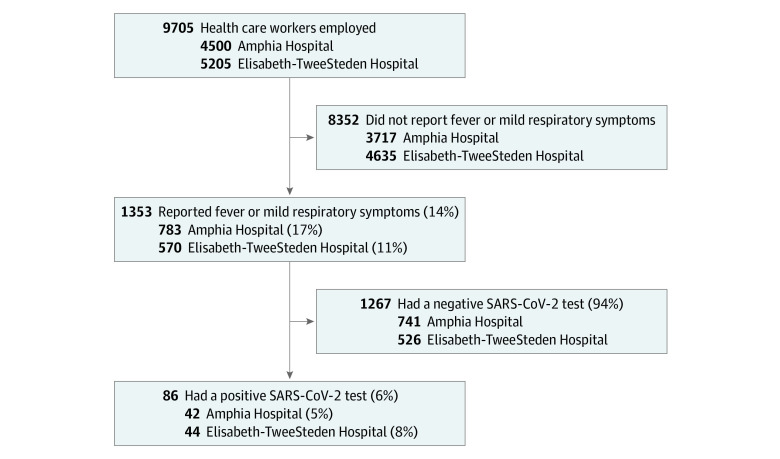
Recruitment of Health Care Workers SARS-CoV-2 indicates severe acute respiratory syndrome coronavirus 2.

A cross-sectional study was conducted in 2 teaching hospitals (700-bed Amphia Hospital, Breda, the Netherlands; 800-bed Elisabeth-TweeSteden Hospital, Tilburg, the Netherlands) employing 9705 HCWs (Figure 1). Between March 7, 2020, and March 12, 2020, HCWs with self-reported fever or (mild) respiratory symptoms in the last 10 days were tested voluntarily for SARS-CoV-2 infection, following the local infection control policy during outbreaks.

### Procedures

A semiquantitative real-time reverse transcriptase–polymerase chain reaction (45 cycles) targeting the SARS-CoV-2 E-gene with high analytical sensitivity and specificity was performed on self-collected oropharyngeal samples, as described previously^4^ and in eAppendix 1 in the Supplement. Structured interviews were conducted between March 12, 2020, and March 16, 2020, to document symptoms for all HCWs with confirmed COVID-19, including those who received a diagnosis before March 7, 2020 (eAppendix 2 in the Supplement). Data were collected with EDC data collection software version 2020.1 (Castor). Recovery was defined as being without symptoms for more than 24 hours.

### Statistical Analysis

Given the descriptive nature of this study, sample size calculations and analysis for statistical significance were not performed. Continuous variables were expressed as medians and ranges. Categorical variables were summarized as counts and percentages. There were no missing data. All analyses were performed with SPSS statistical software version 25.0 (IBM Corp). Data analysis was performed in March 2020.

## Results

**Figure 2.**
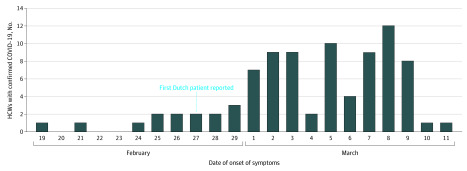
Date of Onset of Symptoms for 86 Health Care Workers (HCWs) With Confirmed Coronavirus Disease 2019 (COVID-19)

Of 9705 HCWs employed (1722 male [18%]), 1353 (14%) reported fever or respiratory symptoms and were tested. Of those, 86 HCWs (6%) were infected with SARS-CoV-2, representing 1% of all HCWs employed (Figure 1). Health care workers with COVID-19 were employed in 52 different hospital departments, including 36 medical wards, and had a median age of 49 years (range, 22-66 years); 15 (17%) were male (Table). Most HCWs with COVID-19 experienced mild disease. Forty-six (53%) HCWs reported fever during the course of illness, and another 10 (12%) reported a feverish feeling without having measured their temperature. Eighty HCWs (93%) met a case definition of fever and/or coughing and/or shortness of breath. Extending this case definition with severe myalgia and/or general malaise would capture all 86 (100%) HCWs with COVID-19 in this evaluation. Other frequent symptoms were headache (49 HCWs [57%]), a runny nose (46 HCWs [53%]), a sore throat (34 HCWs [40%]), chest pain (25 HCWs [29%]), diarrhea (16 HCWs [19%]), and loss of appetite (15 HCWs [17%]). Seven HCWs (8%) indicated that they were already symptomatic before February 27, 2020, the day the first Dutch patient with COVID-19 was diagnosed (Figure 2).

**Table.** Demographic Characteristics, Self-Reported Symptoms, and Outcomes of Health Care Workers With Confirmed Coronavirus Disease 2019

Characteristic	Health care workers, No. (%)		
	Overall (N = 86)	Interview within 7 d of the onset of symptoms (n = 31)	Interview >7 d beyond the onset of symptoms (n = 55)
Demographic characteristics			
Male	15 (17)	6 (19)	9 (16)
Age, median (range), y	49 (22-66)	47 (27-66)	49 (22-65)
Profession			
Physician	12 (14)	2 (6)	10 (18)
Nurse	24 (28)	9 (29)	15 (27)
Other, direct patient contact	29 (34)	12 (39)	17 (31)
Other, no direct patient contact	21 (24)	8 (26)	13 (24)
Self-reported symptoms			
Fever^a^	46 (53)	20 (65)	26 (47)
Feeling feverish, temperature not measured	10 (12)	1 (3)	9 (16)
Coughing	66 (77)	21 (68)	45 (82)
Shortness of breath	33 (38)	6 (19)	27 (49)
Sore throat	34 (40)	11 (35)	23 (42)
Runny nose	46 (53)	17 (55)	29 (53)
General malaise	65 (76)	21 (68)	44 (80)
Severe myalgia	54 (63)	21 (68)	33 (60)
Headache	49 (57)	18 (58)	31 (56)
Chest pain	25 (29)	9 (29)	16 (29)
Abdominal pain	5 (6)	1 (3)	4 (7)
Diarrhea or loose stools	16 (19)	5 (16)	11 (20)
Loss of appetite or nausea	15 (17)	1 (3)	14 (25)
Altered or lost sense of taste	6 (7)	0	6 (11)
Other	17 (20)	2 (6)^b^	15 (27)^c^
Outcomes on the day of the interview			
Recovered	19 (22)	8 (26)	11 (20)
Time until recovery for those recovered, median (range), d	8 (1-20)	5 (1-7)	9 (8-20)
Time until interview for those not recovered, median (range), d	9 (4-25)	6 (4-7)	12 (8-25)
Time since the positive severe acute respiratory syndrome coronavirus 2 test, median (range), d	6 (2-11)	4 (2-6)	6 (2-11)
Hospital admission	2 (2)	0	2 (4)

^a^ Fever was defined as a body temperature of 38.0 °C or higher.

^b^ Other symptoms included painful or burning eyes and painful joints.

^c^ Other symptoms included hoarseness, itchy nose, ear pain, sinus pain, painful or burning eyes, syncope, agitation or palpitation, vomiting, hemoptysis, constipation, and skin rash.

Four HCWs (5%) had recovered by the day of screening and 19 (22%) had recovered by the day of the interview, with a median duration of illness of 8 days (range, 1-20 days) (Table). Two HCWs (2%) were admitted to the hospital and did not develop critical disease up to the moment of reporting. Among HCWs who were interviewed during the second week of illness, coughing (45 HCWs [82%] vs 21 HCWs [68%]), shortness of breath (27 HCWs [49%] vs 6 HCWs [19%]), general malaise (44 HCWs [80%] vs 21 HCWs [68%]), loss of appetite (14 HCWs [25%] vs 1 HCW [3%]), and altered or lost sense of taste (6 HCWs [11%] vs 0 HCWs) were reported more frequently compared with HCWs who were interviewed during the first week of illness. Twenty-one HCWs (24%) had no patient contact during their work, and only 3 (3%) reported having been exposed to an inpatient known to have been diagnosed with COVID-19 before the onset of symptoms. Fifty-four HCWs (63%) mentioned having worked while being symptomatic. Until March 7, 2020, the first day of the HCW screening, 9 patients with documented SARS-CoV-2 infection were hospitalized in the Amphia Hospital and 5 in the Elisabeth-TweeSteden Hospital. Only 3 (3%) of the infected HCWs identified through our screening met the internationally recommended case definition for suspected COVID-19, which included a history of travel to China or northern Italy. When using the definition without travel history to capture community transmission, 44 HCWs (51%) still would not have been detected.

**Figure 3.**
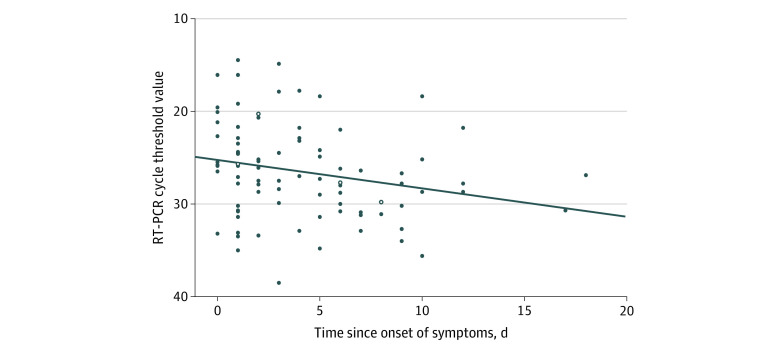
Cycle Threshold Values for the Semiquantitative Reverse Transcriptase–Polymerase Chain Reaction (RT-PCR) (E-gene) by the Time Since the Onset of Symptoms The cycle threshold value is the number of cycles at which the fluorescence exceeds the threshold. Open circles indicate cycle threshold values for health care workers who had recovered by the day of screening.

The median real-time reverse transcriptase–polymerase chain reaction cycle threshold value (ie, the number of cycles at which the fluorescence exceeds the threshold) was 27.0 (range, 14.5-38.5). Within the limited resolution in time since the onset of symptoms, cycle threshold values tended to be higher in HCWs who were tested later in the course of the disease (Figure 3). Cycle threshold values were similar for HCWs with and without self-reported fever on the day of testing (median, 25.1 vs 27.6) and for HCWs with and without any self-reported symptoms on the day of testing (median, 27.0 vs 26.7).

## Discussion

Two weeks after the first Dutch patient with COVID-19 was reported, the prevalence of COVID-19 in HCWs with self-reported fever or respiratory symptoms in 2 Dutch hospitals in the southern part of the Netherlands was 6%, representing 1% of all HCWs employed. This unexpected high prevalence supported the hypothesis of hidden community spread of SARS-CoV-2. Since March 1, 2020, all patients with fever or respiratory symptoms in both hospitals were routinely tested for SARS-CoV-2, and until March 7, 2020, only a limited number of SARS-CoV-2–infected patients were documented, all of whom were nursed under strict isolation precautions. Almost one-quarter (24%) of SARS-CoV-2-infected HCWs had no patient contact at all, and only 3 mentioned exposure to an inpatient known with COVID-19. Although we cannot exclude acquisition from known or unknown SARS-CoV-2–infected patients or HCWs to have occurred in some instances, hospital acquisition is unlikely to explain the vast majority of cases coming from more than 50 different departments in 2 hospitals. The low percentage of men among HCWs with COVID-19 (17%) reflects that among the source population of HCWs in the 2 participating hospitals (18%).

Most HCWs experienced mild disease compared with the clinical presentation and outcomes reported for hospitalized patients so far.^5,6^ Notably, fever or a feverish feeling was frequently not reported. It is still unknown what a sensitive case definition for early detection of SARS-CoV-2–infected individuals would be. At the time of the study, the internationally recommended case definition including a history of travel to China or northern Italy,^1^ applied for only 3 (3%) of the infected HCWs identified through our screening. When using the definition without travel history to capture community transmission, approximately one-half (51%) of HCWs with COVID-19 in our hospitals still would not have been detected. Sensitive detection of COVID-19 cases in HCWs is crucial for hospital infection control policy, particularly for those working with vulnerable patients. We, therefore, suggest adjusting the currently used case definition for suspected COVID-19 in HCWs by taking fever as 1 of the possible symptoms and not as a required symptom. Further improvement of the sensitivity of COVID-19 detection in HCWs can be achieved by adding severe myalgia and general malaise to the case definition. To the best of our knowledge, this report is the first to describe the prevalence and clinical presentations of COVID-19 among HCWs, which may be helpful for others seeking to identify HCWs with suspected COVID-19 in an outbreak situation.

### Limitations

This study has several limitations. First, the screening of HCWs was based on the presence of fever or respiratory symptoms in the last 10 days, and no data were collected for HCWs without these symptoms. The observed 1% prevalence in all HCWs is, thus, a minimal estimate. The lack of data for asymptomatic HCWs also precluded estimates of the sensitivity and specificity of the reported symptoms. Second, oropharyngeal swabs were used for testing, which may have slightly lower sensitivity than a nasopharyngeal swab.^7^ Third, only 22% of SARS-CoV-2–infected HCWs had recovered by the day of the interview, which limits the evaluation of symptoms during the course of the disease. However, stratification of symptoms by time since the onset of symptoms indicated that coughing, shortness of breath, general malaise, loss of appetite, and altered or lost sense of taste were reported more frequently during the second week of illness. Fourth, although the heads of departments and supervisors insisted that HCWs had themselves tested when they had experienced fever or respiratory symptoms in the 10 days before the screening, testing was voluntary and based on self-reported symptoms. This may have led to either overreporting or underreporting of specific symptoms (self-report bias). Overreporting of symptoms would have resulted in the testing of more individuals without SARS-CoV-2 infection and, thus, in underestimation of the prevalence in symptomatic HCWs. Underreporting of symptoms, on the other hand, would have resulted in less testing and underestimation of the prevalence of SARS-CoV-2 infection in the overall group of HCWs. In Dutch hospitals, sick leave has no personal financial consequences. Underreporting is, therefore, not expected to be substantial in this group of professionals with a high sense of responsibility. In addition, recall bias is unlikely to have affected the reporting of fever or respiratory symptoms. Health care workers were not aware of their SARS-CoV-2 infection status at the time of testing, and the recall period was short (up to 10 days). At the time of the interview, however, participants had knowledge of their SARS-CoV-2–positive test, and recall bias could thus have influenced the spectrum of symptoms reported. Prospective studies using diaries and, if possible, documenting symptoms while masking participants for test results may overcome such bias.

## Conclusions

During the containment phase and within 2 weeks after the first Dutch case was detected, a substantial proportion of HCWs with self-reported fever or respiratory symptoms were infected with SARS-CoV-2, likely as the result of acquisition of the virus in the community during the early phase of local spread. This observation confirms the insidious nature of SARS-CoV-2 spread, given the high prevalence of mild clinical presentations that may go undetected.^8^ The spectrum of mild symptoms present in HCWs with COVID-19, frequently not including fever, suggests that the currently recommended case definition for suspected COVID-19 should be used less stringently.

## Notes

Supplement. **eAppendix 1.** Semiquantitative Real-Time Reverse-Transcriptase PCR for SARS-CoV-2 **eReferences.** **eAppendix 2.** Questionnaire Used for Interviewing Health Care Workers With COVID-19
