# Supplementary material for: Prevalence and Clinical Presentation of Health Care Workers With Symptoms of Coronavirus Disease 2019 in 2 Dutch Hospitals During an Early Phase of the Pandemic
Source: JAMA Netw Open. 2020 May 21;3(5):e209673. doi: 10.1001/jamanetworkopen.2020.9673 (PMC7243090; doi:10.1001/jamanetworkopen.2020.9673)
Supplement: Supplement. — eAppendix 1. Semiquantitative Real-Time Reverse-Transcriptase PCR for SARS-CoV-2 eReferences. eAppendix 2. Questionnaire Used for Interviewing Health Care Workers With COVID-19 [file jamanetwopen-3-e209673-s001.pdf]

Supplementary Online Content

Kluytmans-van den Bergh MFQ, Buiting AGM, Pas SD, et al. Prevalence and clinical presentation of health care workers with symptoms of coronavirus disease 2019 in 2 Dutch hospitals during an early phase of the pandemic. *JAMA Netw Open*. 2020;3(5):e209673. doi:10.1001/jamanetworkopen.2020.9673

eAppendix 1. Semiquantitative Real-Time Reverse-Transcriptase PCR for SARS-CoV-2

eReferences

eAppendix 2. Questionnaire Used for Interviewing Health Care Workers With COVID-19

This supplementary material has been provided by the authors to give readers additional information about their work.

© 2020 Kluytmans-van den Bergh MFQ et al. *JAMA Network Open.*

eAppendix 1. Semiquantitative Real-Time Reverse-Transcriptase PCR for SARS-CoV-2

After an external lysis step (1:1 with lysis/binding buffer (Roche Diagnostics, the Netherlands), total nucleic acids were extracted using MagnaPure96 (Roche) with an input volume of 500 µl and output volume of 100 µl. The extraction was internally controlled by the addition of a known concentration of phocine distemper virus (PDV).[1] Subsequently 10 µl extracted nucleic acids was amplified in three singleplex reactions 25 µl final volume, using TaqMan Fast Virus 1-Step Master Mix (Thermofisher, Nieuwerkerk a/d IJssel, the Netherlands), and 1 µl of primers and probe mixture for E gene, RdRP gen as described previously.[2] Amplification was performed in a 7500SDS (Thermofisher) with a cycling profile of 5 min at 50°C, 20 s at 95°C, 45 cycles of 3 s at 95°C and 30 s at 58°C. Alternatively, total nucleic acids were extracted, with a known concentration of PDV as internal control, using the QIAsymphony DSP virus/pathogen midi kit and pathogen complex 400 protocol of the QIAsymphony Sample Processing (SP) system (Qiagen, Hilden, Germany) with an input volume 400 µl and output volume of 110 µl. Amplification reactions were performed in a volume of 25 µL with TaqMan® Fast Virus 1-Step Master Mix (Thermofisher) and 10 µL extracted nucleic acids. A duplex PCR for E-gen/PDV and if positive a duplex PCR for RdRP/PDV with optimized primer and probe concentrations were performed.[1,3] Amplification using Rotorgene (QIAgen) consisted of 5 min at 50°C, 15 min at 95°C followed by 45 cycles of 15 s at 95°C, 30 s at 60°C, and 15s at 72°C. Validations of RT-PCR procedures were performed according to International Standards Organization guidelines 15189 (http://www.iso.org/iso/search.htm).
Analytical sensitivity was tested using a sensitivity panel (kindly provided by the National Institute for Public Health and the Environment (RIVM), the Netherlands), in which 0.83 copies/ml (~5.62E-2 TCID50/ml) were still detectable. Analytical specificity was tested on 22 high-titred stocks (median Ct value 19.4, range 10.6-32.4) from known pathogens causing respiratory disease (HCoV-229E, HCoV-OC43, HCoV-NL63, MERS-CoV, influenza A virus, influenza B virus, human rhinovirus, human metapneumovirus, respiratory syncytial virus type A and B, parainfluenza virus type 1-4, bocavirus, adenovirus types 1, 7 and 40, *Mycoplasma pneumoniae*, *Chlamydia psittaci*, *Chlamydophila pneumoniae*, *Legionella pneumoniae*) and 50 clinical respiratory samples (oropharyngeal swabs, bronchoalveolar lavages, sputa), in all of which SARS-CoV-2 RNA was undetectable.

eReferences

1. Hoek RAS, Paats MS, Pas SD, et al. Incidence of viral respiratory pathogens causing exacerbations in adult cystic fibrosis patients. *Scand J Infect Dis*. 2013;45 (1):65-9. doi: 10.1016/j.jcf.2013.05.002.
2. Corman VM, Landt O, Kaiser M, et al. Detection of 2019 novel coronavirus (2019-nCoV) by real-time RT-PCR. *Euro Surveill*. 2020;25(3):pii=2000045. doi:10.2807/1560-7917.ES.2020.25.3.2000045.
3. van der Vries E, Anber J, van der Linden A, et al. Molecular assays for quantitative and qualitative detection of influenza virus and oseltamivir resistance mutations. *J Mol Diagn.* 2013;15(3):347-54. doi:10.1016/j.jmoldx.2012.11.007.

© 2020 Kluytmans-van den Bergh MFQ et al. *JAMA Network Open.*

eAppendix 2. Questionnaire Used for Interviewing Health Care Workers With COVID-19.

1. Hospital
2. Department
3. Profession
4. Sex
5. Year of birth
6. Symptoms on day of SARS-CoV-2 testing
    a. Fever (>=38ºC)
    b. Coughing
    c. Sore throat
    d. Runny nose
    e. (Severe) myalgia
    f. General malaise
    g. Headache
    h. Shortness of breath
    i. Chest pain
    j. Abdominal pain
    k. Diarrhea
    l. Other, please specify
7. Symptoms in 10 days before the day of SARS-CoV-2 testing
    a. Fever (>=38ºC)
    b. Coughing
    c. Sore throat
    d. Runny nose
    e. (Severe) myalgia
    f. General malaise
    g. Headache
    h. Shortness of breath
    i. Chest pain
    j. Abdominal pain
    k. Diarrhea
    l. Other, please specify
8. Symptoms between day of SARS-CoV-2 testing until day of interview
    a. Fever (>=38ºC)
    b. Coughing
    c. Sore throat
    d. Runny nose
    e. (Severe) myalgia
    f. General malaise
    g. Headache
    h. Shortness of breath
    i. Chest pain
    j. Abdominal pain
    k. Diarrhea
    l. Other, please specify
9. Hospital admission
10. Having worked while being symptomatic
11. First day of symptoms
12. Last day of symptoms (if recovered)
13. Day of SARS-CoV-2 test
14. Day of interview

© 2020 Kluytmans-van den Bergh MFQ et al. *JAMA Network Open.*
